# Integrin α5β1 expression on dopaminergic neurons is involved in dopaminergic neurite outgrowth on striatal neurons

**DOI:** 10.1038/srep42111

**Published:** 2017-02-08

**Authors:** Yasuhiko Izumi, Seiko Wakita, Chisato Kanbara, Toshie Nakai, Akinori Akaike, Toshiaki Kume

**Affiliations:** 1 Department of Pharmacology, Graduate School of Pharmaceutical Sciences, Kyoto University, 46-29 Shimoadachi-cho, Sakyo-ku, Kyoto 606-8501, Japan; 2 Department of Cellular Pharmacology, Graduate School of Pharmaceutical Sciences, Nagoya University, Furo-cho, Chikusa-ku, Nagoya 464-8601, Japan

## Abstract

During development, dopaminergic neurons born in the substantia nigra extend their axons toward the striatum. However, the mechanisms by which the dopaminergic axons extend the striatum to innervate their targets remain unclear. We previously showed that paired-cultivation of mesencephalic cells containing dopaminergic neurons with striatal cells leads to the extension of dopaminergic neurites from the mesencephalic cell region to the striatal cell region. The present study shows that dopaminergic neurites extended along striatal neurons in the paired-cultures of mesencephalic cells with striatal cells. The extension of dopaminergic neurites was suppressed by the pharmacological inhibition of integrin α5β1. Using lentiviral vectors, short hairpin RNA (shRNA)-mediated knockdown of integrin α5 in dopaminergic neurons suppressed the neurite outgrowth to the striatal cell region. In contrast, the knockdown of integrin α5 in non-dopaminergic mesencephalic and striatal cells had no effect. Furthermore, overexpression of integrin α5 in dopaminergic neurons differentiated from embryonic stem cells enhanced their neurite outgrowth on striatal cells. These results indicate that integrin α5β1 expression on dopaminergic neurons plays an important role in the dopaminergic neurite outgrowth on striatal neurons.

Dopaminergic neurons in the substantia nigra pars compacta project to the dorsolateral striatum, thus forming the nigrostriatal projection. In humans, a selective loss of this projection is a pathological hallmark of Parkinson disease (PD). Although the exact causes of neuronal loss remain unclear, the regeneration of this pathway shows great promise as a therapy for PD. Transplantation of fetal nigral dopamine neurons for PD patients gives rise to substantial symptomatic relief for a decade^1^, although it have been reported that dyskinesia occurs after transplantation^2^. Olanow *et al*.^3^ suggest that it is likely that off-medication dyskinesia represent a variant of diphasic dyskinesia due to incomplete or aberrant reinnervation of the striatum. To successfully reestablish the lost nigrostriatal pathway, considerable knowledge regarding the axon guidance signals present in the extracellular milieu is required.

In rat and mouse embryos, dopaminergic axons, which begin their projection in a dorsal direction from the ventrocaudal region of the midbrain, are rostrally guided by Wnt5a and Semaphorin 3F as chemorepellants and Wnt7b as a chemoattractant^4^,^5^,^6^,^7^. The dopaminergic axons rostrally elongate along the medial forebrain bundle as they move ventrally to the thalamus. Next, the axons are directed into the striatum via the repulsive effect of ephrin-A5^8^. Although a correlation between dopaminergic innervation and the striatal distribution of glycosaminoglycans during the early postnatal period has been suggested^9^, it remains unclear how the dopaminergic axons extend to the striatum in order to innervate their targets.

To address this issue, we previously reconstructed the dopaminergic innervation of striatal cells using dissociated primary cultures^10^. When mesencephalic cells containing dopaminergic neurons were adjacently paired-cultured with striatal cells, dopaminergic neurites extended from the mesencephalic cell region to the striatal cell region. In addition, this paired-cultivation enables the quantitative evaluation of dopaminergic neurite outgrowth to the striatal cell region. In the present study, we pharmacologically and genetically examined the precise mechanisms behind the dopaminergic neurite outgrowth on striatal neurons in the paired-cultures. Here, we show that integrin α5β1 expression on dopaminergic neurons is involved in the dopaminergic neurite outgrowth on striatal neurons. In addition, we investigated the neurite outgrowth of integrin α5-overexpressing dopaminergic neurons derived from embryonic stem (ES) cells on striatal cultures.

## Results

### Dopaminergic neurite outgrowth on striatal neurons in paired-cultures of mesencephalic and striatal cells

**Figure 1:**
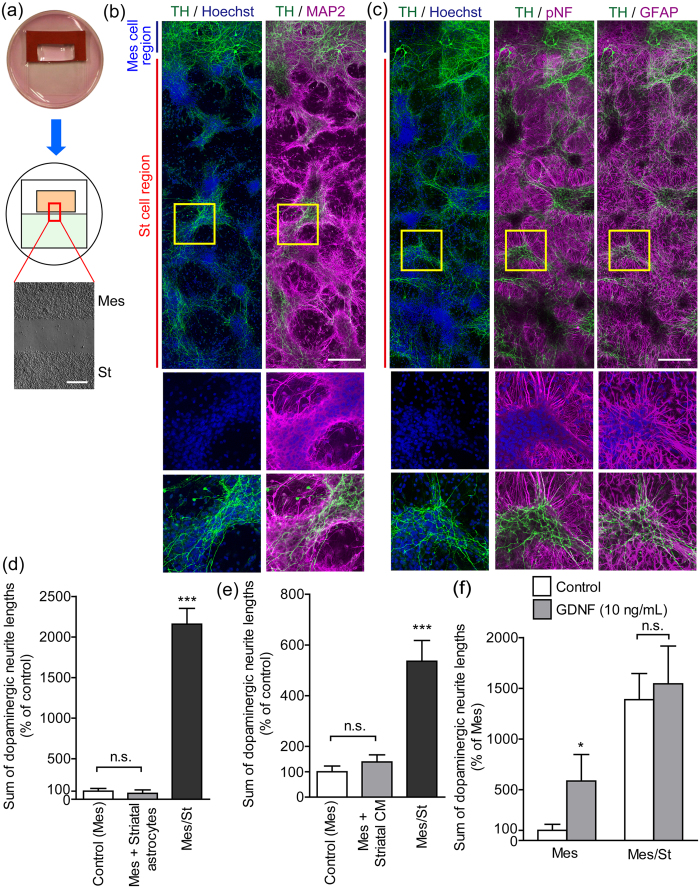
The effects of striatal cells on dopaminergic neurite outgrowth in paired-cultures of mesencephalic and striatal cells. (**a**) Scheme of paired-cultures of mesencephalic and striatal cells. A coverslip in a 35 mm culture dish was divided into two compartments with an isolation wall. Mesencephalic cells were plated inside the isolation wall and striatal cells were plated outside. The isolation wall was removed 24 h after plating. A microphotograph shows the vicinity of the borderline. The upper region is comprised of mesencephalic cells (Mes), and the lower region is comprised of striatal cells (St). Scale bar = 200 μm. (**b** and **c**) Representative photographs of the dopaminergic innervation of striatal cells. Mesencephalic cells were paired-cultured with striatal cells for 10 days, and the paired-cultures were processed for TH, MAP2, pNF, and GFAP immunostaining and nuclear staining. Fluorescence images show the striatal cell region near the borderline. The lower images were high-power fields outlined by a square in the upper images. Scale bar = 200 μm. (**d**) Striatal astrocytes had no effect on dopaminergic neurite outgrowth. Control (Mes) group: mesencephalic cells were cultured alone for 10 days. Mes + Striatal astrocytes group: mesencephalic cells were paired-cultured with astrocytes derived from the striatum. Mes/St group: mesencephalic cells were paired-cultured with striatal cells. (**e**) Striatal CM did not affect dopaminergic neurite outgrowth. Mes + Striatal CM group: mesencephalic cells were cultured in CM prepared from striatal cultures. (**f**) GDNF did not enhance dopaminergic neurite outgrowth in paired-cultures of mesencephalic and striatal cells. Mesencephalic cells only or paired-cultures of mesencephalic and striatal cells were incubated in the presence or absence of GDNF (10 ng/mL) for 10 days. *p < 0.05 and ***p < 0.001 vs. control (Mes) group. n.s.: not significant.

Our previous study^10^ demonstrated that dopaminergic neurites extended from the mesencephalic cell region to the striatal cell region after 10 days of paired-cultivation of mesencephalic and striatal cells (Fig. 1a). We used fluorescence staining to clarify the pattern of dopaminergic neurite outgrowth to the striatal cell region. Nuclear staining with Hoechst 33258 in the striatal cell region revealed that striatal cells formed clusters after 10 days in culture. The clusters of striatal cells were surrounded by microtubule associated protein 2 (MAP2)-positive dendrites of striatal neurons, suggesting that striatal neurons tended to form clusters (Fig. 1b). Phosphorylated neurofilaments (pNF)-positive axons of striatal neurons connected the clusters (Fig. 1c). Importantly, tyrosine hydroxylase (TH)-positive dopaminergic neurites extended along the clusters of striatal neurons (Fig. 1b and c). Glial fibrillary acidic protein (GFAP)-positive astrocytes were mainly localized to the striatal cell region outside of the clusters (Fig. 1c). In our previous report^10^, synaptophysin was expressed at the growth cones of dopaminergic neurites which extended along the striatal neuronal clusters. To examine the involvement of striatal astrocytes in dopaminergic neurite outgrowth, mesencephalic cells were adjacently paired-cultured with striatal astrocytes for 10 days. The paired-cultivation with striatal astrocytes had no effect on dopaminergic neurite outgrowth (Fig. 1d). To examine the involvement of humoral factors from striatal cells in dopaminergic neurite outgrowth, mesencephalic cells were cultured in striatal cell-conditioned medium (CM) for 10 days. The striatal CM had no effect on dopaminergic neurite outgrowth (Fig. 1e). We previously reported that glial derived neurotrophic factor (GDNF) extended dopaminergic neurites beyond the mesencephalic cell region in mesencephalic cell cultures^10^. However, GDNF did not enhance dopaminergic neurite outgrowth to the striatal cell region in the paired-cultures of mesencephalic and striatal cells (Fig. 1f), suggesting that the signaling of GDNF was saturated.

### Inhibition of integrin α5β1 suppressed dopaminergic neurite outgrowth to the striatal cell region

**Figure 2:**
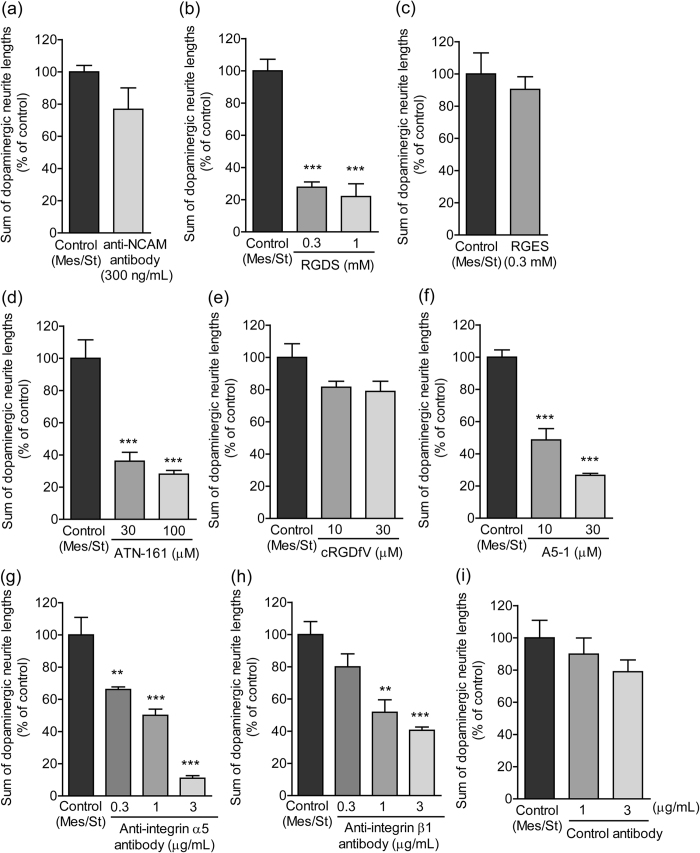
Involvement of cell adhesion molecules in dopaminergic neurite outgrowth to the striatal cell region. Mesencephalic cells were paired-cultured with striatal cells for 10 days in the presence or absence of function-blocking peptides and antibodies. **p < 0.01 and ***p < 0.001 vs. control (Mes/St) group.

Cell adhesion molecules, such as neural cell adhesion molecule (NCAM) and integrin, play important roles in the signaling of GDNF for dopaminergic neurite outgrowth^11^,^12^,^13^. The function-blocking antibody anti-NCAM (AB5032), which inhibited GDNF-induced dopaminergic neurite outgrowth (data not shown), had no effect on the dopaminergic neurite outgrowth to the striatal cell region in the paired-cultures of mesencephalic and striatal cells (Fig. 2a). In contrast, the synthetic Arg-Gly-Asp-Ser (RGDS) peptide, an inhibitor of RGD-binding integrins, significantly suppressed neurite outgrowth, whereas treatment with a glutamic acid-substituted control peptide (RGES) showed no effect (Fig. 2b and c). To narrow down the candidates of integrin heterodimers, the effects of more selective blocking peptides were examined. ATN-161 (Ac-PHSCN-NH_2_), which is derived from the synergy region of fibronectin, binds to both α5β1 and αVβ3 *in vitro*^14^. Cyclo(RGDfV), a cyclic pentapeptide containing the RGD sequence, is a selective αVβ3 antagonist^15^. A5-1 (VILVLF) is an antagonistic peptide against integrin α5β1^16^. ATN-161 and A5-1 significantly suppressed dopaminergic neurite outgrowth to the striatal cell region, whereas treatment with cyclo(RGDfV) had no effect (Fig. 2d–f). To confirm the involvement of integrin α5β1, the effects of function-blocking antibodies were examined. Anti-integrin α5 (HMa5-1) and anti-integrin β1 (Ha2/5) antibodies have function-blocking properties^17^,^18^. These antibodies significantly suppressed dopaminergic neurite outgrowth to the striatal cell region, whereas treatment with control IgG had no effect (Fig. 2g–i).

### Expression of integrin α5β1 in dopaminergic neurons

**Figure 3:**
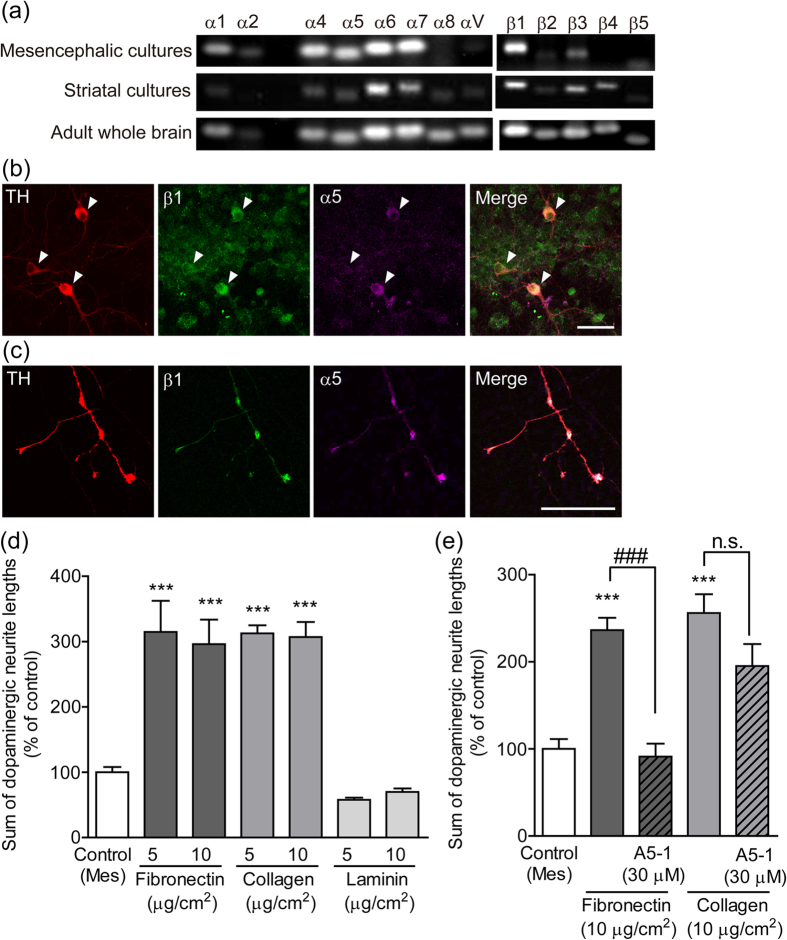
Participation of integrin α5β1 in dopaminergic neurite outgrowth. (**a**) Expression of integrin mRNA in mesencephalic and striatal cultures. mRNA levels were analyzed using RT-PCR. (**b** and **c**) Expression of integrin α5 and β1 on dopaminergic neurons. Representative photographs of dopaminergic soma (**b**) and neurites (**c**). Mesencephalic cells were cultured alone for 10 days and then processed for immunostaining of TH, integrin α5 and integrin β1. Arrowheads indicate dopaminergic soma. Scale bars = 50 μm (**b**) and 25 μm (**c**). (**d**) Influence of the extracellular matrix on dopaminergic neurite outgrowth. The outside of the isolation wall was coated by fibronectin, collagen type I, or laminin (5 and 10 μg/cm^2^). Mesencephalic cells were cultured alone for 10 days. (**e**) The effect of integrin α5β1 blocking peptide on dopaminergic neurite outgrowth on the extracellular matrix. The outside of the isolation wall was coated by fibronectin or collagen type I (10 μg/cm^2^). Mesencephalic cells alone were cultured in the presence or absence of A5-1 (30 μM) for 10 days. ***p < 0.001 vs. control (Mes) group. ^###^p < 0.001. n.s.: not significant.

We examined the expression of integrin mRNA (integrin α1, 2, 4, 5, 6, 7, 8, V, and β1, 2, 3, 4, 5) in mesencephalic and striatal cultures. Whole-brain samples from adult rats were used as a positive control. Although there were differences in expression levels, all of the integrin mRNAs tested in this study were expressed, except for integrin β4 in mesencephalic cultures and integrin α2 in striatal cultures (Fig. 3a). Integrin α5 and β1 were expressed in dopaminergic soma (Fig. 3b). Furthermore, integrin α5 and β1 were intensely expressed in dopaminergic growth cones and varicosities (Fig. 3c). The percentage of dopaminergic neurons expressing integrin α5 and β1 was 96.7 ± 1.9% and 93.3 ± 2.1%, respectively. To examine the involvement of integrin α5β1 in dopaminergic neurite outgrowth, the outside of the mesencephalic cell region was coated with extracellular matrix. Fibronectin is a glycoprotein that binds to integrin α5β1.The fibronectin coating outside of the mesencephalic cell region enhanced dopaminergic neurite outgrowth in mesencephalic cell cultures. Collagen and laminin are extracellular matrices that bind to integrin family members other than integrin α5β1. The collagen type I coating enhanced dopaminergic neurite outgrowth, whereas the laminin coating did not (Fig. 3d). A5-1 suppressed dopaminergic neurite outgrowth on fibronectin coating, but did not on collagen coating (Fig. 3e).

### Knockdown of integrin α5 in mesencephalic cells suppressed dopaminergic neurite outgrowth to the striatal cell region

**Figure 4:**
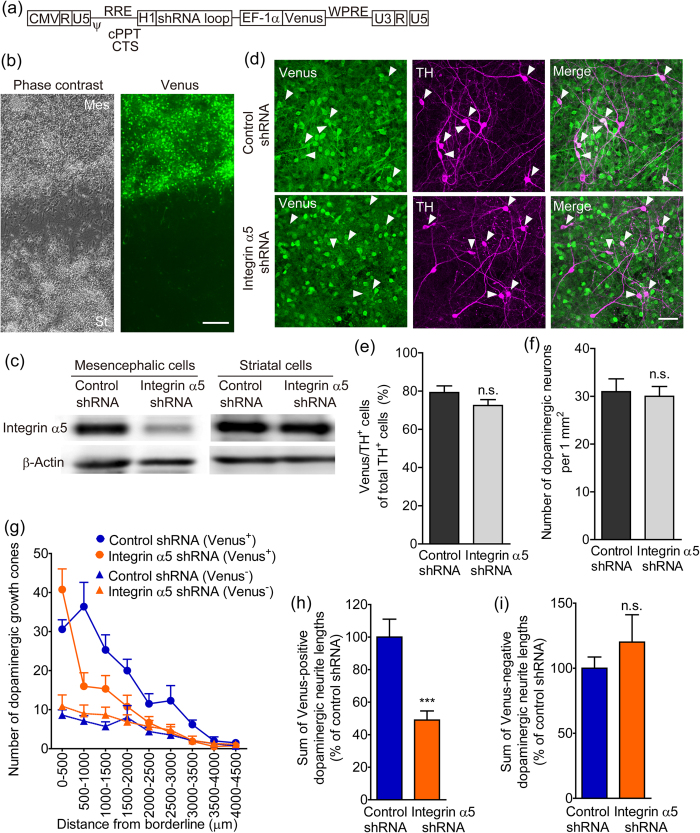
The effect of integrin α5 knockdown in mesencephalic cells on dopaminergic neurite outgrowth to the striatal cell region. (**a**) Structure of the lentiviral vector expressing shRNA and Venus under the control of the human H1 and human EF-1α promoters, respectively. (**b**) Preferential infection of mesencephalic cells with lentiviral vectors. Lentiviral vectors were applied inside the isolation wall during 5 to 24 hours after cell plating. Mesencephalic cells were paired-cultured with striatal cells for 5 days. Representative photographs show the vicinity of the borderline between the cell regions. Left: phase-contrast image. Right: Venus fluorescence. Scale bar = 200 μm. (**c**) Selective knockdown of integrin α5 in mesencephalic cells. Protein samples were separately collected from the mesencephalic and striatal cells of paired-cultures 6 days after infection with lentiviral vectors. β-Actin was used as a loading control. (**d–f**) The effects of lentiviral vectors on infection efficiency and cell density in dopaminergic neurons. After infection with lentiviral vectors, mesencephalic cells were paired-cultured with striatal cells for 10 days and then processed for immunostaining of Venus and TH. Arrowheads (**d**) indicate Venus-positive dopaminergic neurons. Scale bar = 50 μm. n = 20. (**g**) Distribution of Venus-positive and Venus-negative dopaminergic growth cones from the borderline of the mesencephalic cell region. (**h** and **i**) The effect of integrin α5 shRNA infection in mesencephalic cells on dopaminergic neurite outgrowth to the striatal cell region. Venus-positive (**h**) and Venus-negative (**i**) dopaminergic neurite lengths were summed. ***p < 0.001 vs. control shRNA. n.s.: not significant.

Integrin α5β1 was expressed in both mesencephalic and striatal cultures. To clarify the involvement of integrin α5β1 expression on dopaminergic neurons in neurite outgrowth, we completed a lentiviral vector–mediated gene knockdown in mesencephalic cells. The silencing lentiviral vector constructs expressed both shRNA and Venus, which was as a marker gene (Fig. 4a). To produce mesencephalic cell-specific knockdown, the lentiviral vectors were applied to the inside of the isolation wall containing mesencephalic cells. The isolation wall was removed the next day after the lentiviral vectors were washed out. After paired-cultivation with striatal cells for 5 days, Venus was preferentially expressed in mesencephalic cells (Fig. 4b). Integrin α5 shRNA selectively decreased the expression of endogenous integrin α5 in mesencephalic cells and had no detectable effect on the expression in striatal cells (Fig. 4c). After paired-cultivation with striatal cells for 10 days, immunocytochemistry was performed using anti-green fluorescent protein (GFP) (for Venus) and anti-TH antibodies. Both lentiviral vectors, which expressed either control shRNA or integrin α5 shRNA, infected approximately 70% of the dopaminergic neurons (Fig. 4d and e). There was no difference in dopaminergic cell viability between vectors (Fig. 4f). Figure 4g shows that distribution of dopaminergic growth cones from the borderline of the mesencephalic cell region. In cultures transfected with integrin α5 shRNA, the number of Venus-positive dopaminergic growth cones was decreased away from the borderline. Thus, transfection of mesencephalic cells with integrin α5 shRNA significantly suppressed Venus-positive dopaminergic neurite outgrowth to the striatal cell region (Fig. 4h). There was no difference in the distribution of Venus-negative dopaminergic growth cones or the Venus-negative dopaminergic neurite outgrowth between control and integrin α5 shRNA (Fig. 4g and i).

### Knockdown of integrin α5 in striatal cells did not affect dopaminergic neurite outgrowth to the striatal cell region

**Figure 5:**
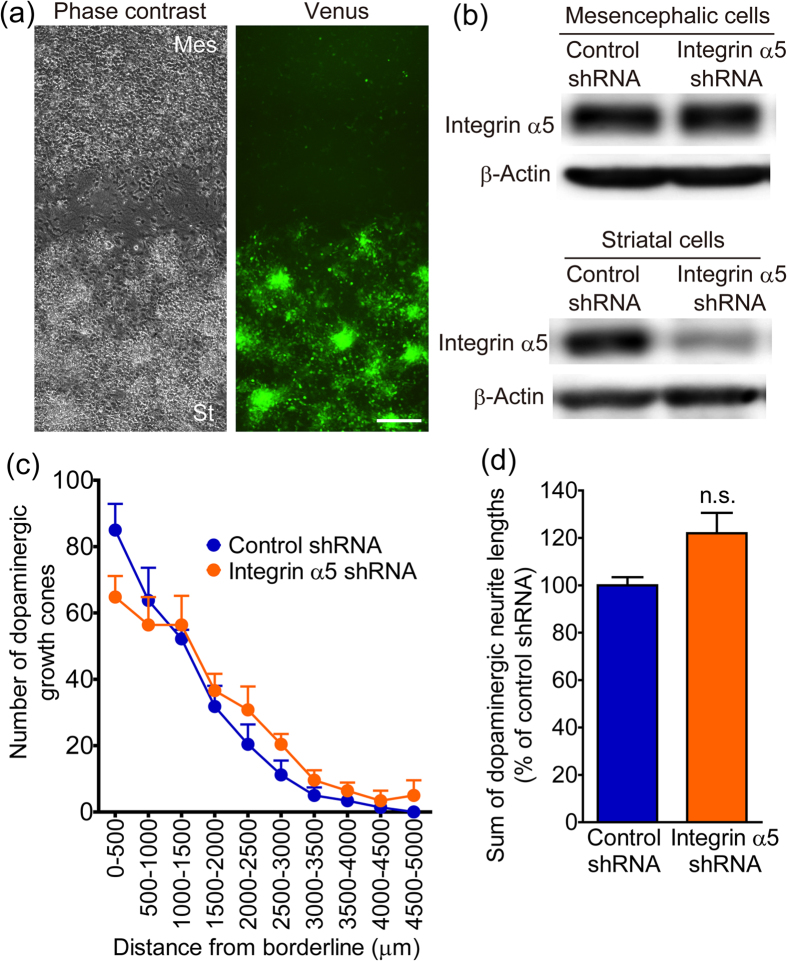
The effect of integrin α5 knockdown in striatal cells on dopaminergic neurite outgrowth to the striatal cell region. (**a**) Preferential infection of striatal cells with lentiviral vectors. Lentiviral vectors were applied outside the isolation wall during 5 to 24 hours after cell plating. Mesencephalic cells were paired-cultured with striatal cells for 5 days. Representative photographs show the vicinity of the borderline between the cell regions. Left: phase-contrast image. Right: Venus fluorescence. Scale bar = 200 μm. (**b**) Selective knockdown of integrin α5 in striatal cells. Protein samples were separately collected from the mesencephalic and striatal cells of paired-cultures 6 days after infection with lentiviral vectors. (**c**) Distribution of dopaminergic growth cones from the borderline of the mesencephalic cell region. (**d**) The effect integrin α5 shRNA infection in striatal cells with on dopaminergic neurite outgrowth to the striatal cell region. After infection with lentiviral vectors, mesencephalic cells were paired-cultured with striatal cells for 10 days and then processed for TH immunostaining. TH-positive dopaminergic neurite lengths were summed. n.s.: not significant.

To eliminate the possibility that integrin α5β1 expression in striatal cells is involved in dopaminergic neurite outgrowth, we selectively knock downed integrin α5 in striatal cells. The lentiviral vectors were applied to the outside of the isolation wall containing striatal cells. After paired-cultivation with striatal cells for 5 days, Venus was preferentially expressed in striatal cells (Fig. 5a). Integrin α5 shRNA selectively decreased the expression of endogenous integrin α5 in striatal cells and had no detectable effect in mesencephalic cells (Fig. 5b). After paired-cultivation with striatal cells for 10 days, immunocytochemistry was performed using anti-TH antibodies. There was no difference in the distribution of dopaminergic growth cones or the dopaminergic neurite outgrowth between control and integrin α5 shRNA (Fig. 5c and d).

### Overexpression of integrin α5 in ES cell-derived dopaminergic neurons enhanced dopaminergic neurite outgrowth on striatal cultures

**Figure 6:**
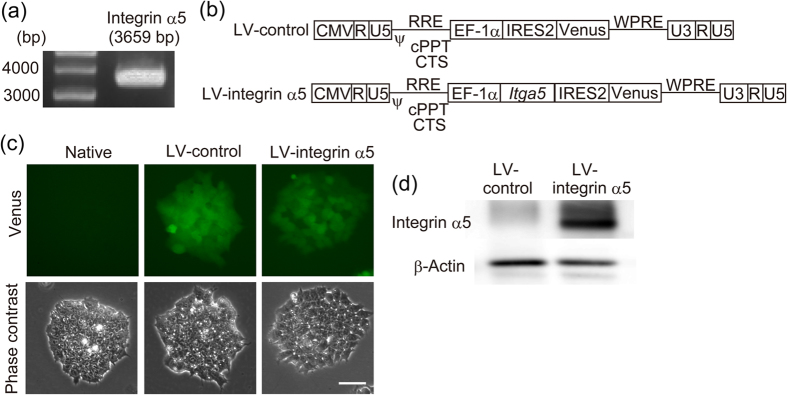
Transduction of integrin α5 in undifferentiated ES cells by lentiviral vectors. (**a**) Full-length mouse integrin α5 mRNA was isolated from whole brains. Integrin α5 cDNA was amplified with PCR using specific primers. (**b**) Structure of the lentiviral vector expressing integrin α5 and/or Venus under the control of the EF-1α promoter. The integrin α5 gene (*Itga5*) was incorporated upstream of the internal ribosome entry site 2 (IRES2) region. The translation of Venus was initiated via IRES2. (**c,d**) Expression of Venus and integrin α5 in undifferentiated ES cells. Mouse ES cells were transfected with lentiviral vectors, and a fluorescent colony of ES cells was picked under the microscope to obtain a single clone. Scale bar = 50 μm.

**Figure 7:**
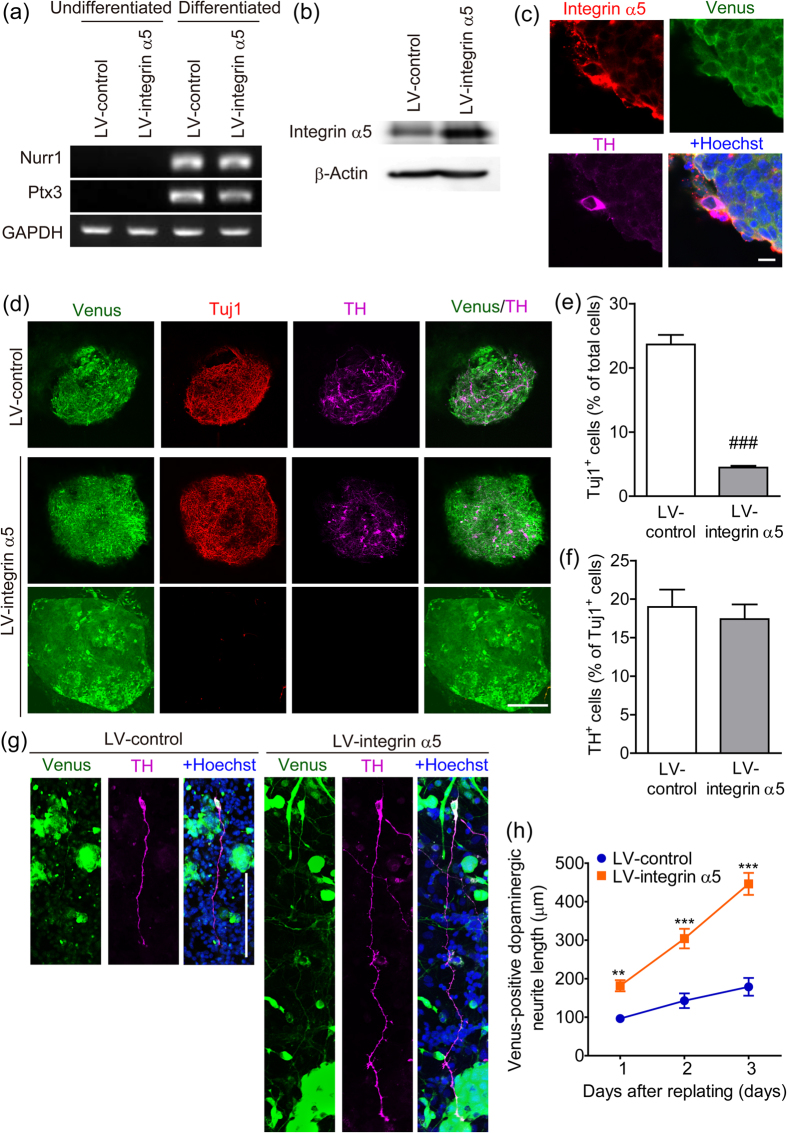
The effect of integrin α5 overexpression on the neurite outgrowth of ES cell-derived dopaminergic neurons on striatal cultures. (**a**) Expression of Nurr1 and Ptx3 mRNA during differentiation of ES cells. ES cells transfected with lentiviral vectors were differentiated with the SDIA method for 12 days. (**b**) Expression of integrin α5 in differentiated ES cells. ES cells transfected with lentiviral vectors were differentiated with the SDIA method for 14 days. (**c**) Integrin α5 expression in TH-positive cells differentiated from LV-control transfected ES cells. Scale bar = 10 μm. (**d**) Venus expression in TH-positive cells differentiated from transfected ES cells. The lower images show a TuJ1-negative colony from LV-integrin α5 transfected cells. Scale bar = 200 μm. (**e**) The effect of integrin α5 overexpression on neural differentiation efficiency of ES cells. ES cell-derived colonies were detached from feeder cells after 14 days of differentiation and then dissociated into single cells. These cells were processed for Venus and β-III tubulin (TuJ1) immunostaining and analyzed by flow cytometry. (**f**) The effect of integrin α5 overexpression on dopaminergic differentiation efficiency of ES cells. (**g** and **h**) The effect of integrin α5 overexpression on the neurite length of ES cell-derived dopaminergic neurons. ES cell-derived colonies were detached from feeder cells after 13 days of differentiation and then dissociated into single cells. These cells were replated on striatal cultures and then processed for immunostaining after cultivation for the indicated times. Representative images (**g**) were obtained 2 days after cultivation on striatal cultures. Scale bar = 100 μm. n = 10–18. **p < 0.01 and ***p < 0.001 vs. LV-control.

To confirm that integrin α5β1 expression on dopaminergic neurons participates in the neurite outgrowth on striatal neurons, we produced integrin α5-overexpressing dopaminergic neurons from mouse ES cells. Full-length mouse integrin α5 mRNA was isolated from the whole brain (Fig. 6a). cDNA was incorporated into a bicistronic IRES-Venus expression lentiviral vector (LV-integrin α5). The control vector (LV-control) consisted of the lentiviral backbone vector with the gene encoding Venus (Fig. 6b). Mouse ES cells were transfected with these lentiviral vectors and cloned. We confirmed that undifferentiated ES cells transfected with the vectors expressed Venus (Fig. 6c), LV-integrin α5-transfected cells expressed integrin α5, and undifferentiated ES cells did not express endogenous integrin α5 (Fig. 6d). The cloned ES cells were differentiated with the stromal cell-derived inducing activity method (SDIA method), by which mesencephalic dopaminergic neurons are induced^19^. We confirmed that the mesencephalic dopaminergic neuron markers *Nurr1* and *Ptx3* were induced in differentiated ES cells (Fig. 7a). After differentiation, LV-integrin α5-transfected cells expressed more integrin α5 than LV-control-transfected cells (Fig. 7b). Confocal microscopy showed that integrin α5 was expressed at the plasma membrane of dopaminergic neurons differentiated from LV-control-transfected ES cells (Fig. 7c). We confirmed that Venus expression in TH-positive neurons was maintained in both clones. Although more than 90% of colonies from LV-control-transfected cells were positive for neuron-specific β-III tubulin (TuJ1), more than 50% of colonies from LV-integrin α5-transfected cells were negative for TuJ1 (Fig. 7d). Flow cytometry demonstrated that the proportion of TuJ1-positive cells to total ES cells was significantly decreased by transfection with LV-integrin α5 (Fig. 7e). Regardless of the inefficiency of neural differentiation, immunofluorescence double-staining demonstrated that the proportion of TH-positive cells to TuJ1-positive cells was similar between LV-control- and LV-integrin α5- transfected cells (Fig. 7f). To examine the effect of integrin α5 overexpression on dopaminergic neurite outgrowth, ES cell-derived dopaminergic neurons were replated on striatal cultures. We measured the total neurite lengths of Venus-positive dopaminergic neurons, and overexpression of integrin α5 in dopaminergic neurons enhanced dopaminergic neurite outgrowth on striatal cultures (Fig. 7g). In addition, the difference between LV-control- and LV-integrin α5- transfected groups in dopaminergic neurite outgrowth expanded over time (1–3 days) (Fig. 7h).

## Discussion

In this study, we demonstrated that both pharmacological and genetic inhibition of integrin α5β1 suppressed the dopaminergic neurite outgrowth on striatal neurons. Furthermore, we showed that overexpression of integrin α5 enhanced the neurite outgrowth of stem cell-derived dopaminergic neurons on striatal cells. These findings suggest that integrin α5β1 expression on dopaminergic neurons plays an important role in the dopaminergic neurite outgrowth on striatal neurons.

Our previous report showed that in paired-cultivation conditions, dopaminergic axons prefer to extend to the striatal cell region rather than the spinal cell region^10^. Diffusible and/or membrane-bound factors derived from striatal cells enhance the maturation of dopaminergic neurons^20^,^21^. In organotypic co-cultures of mesencephalic and striatal slices, the dopaminergic innervation of striatal slices is thought to result from adhesive interactions rather than from diffusible substances^22^. However, the axon-promoting factors involved in the dopaminergic neurite outgrowth on striatal neurons remain unknown. In paired-cultures with striatal cells, mesencephalic dopaminergic neurites extended along striatal neurons but not striatal astrocytes. This observation indicates that cell adhesion molecules between dopaminergic neurites and striatal neurons are important for the dopaminergic neurite outgrowth on striatal neurons. In contrast, CM derived from striatal cultures did not enhance dopaminergic neurite outgrowth, suggesting a small contribution of humoral factors to dopaminergic neurite outgrowth in our cultures. Because the concentration gradients of chemoattractants are important for the directional guidance of neurite outgrowth^23^, the exchange for CM derived from striatal cultures may be insufficient for the outward extension of dopaminergic neurites from the mesencephalic cell region. Interestingly, GDNF, which enhanced the outward extension of dopaminergic neurites from the mesencephalic cell region in mesencephalic cells only cultures, had no effect in paired-cultures of mesencephalic and striatal cells. This finding suggests that the interaction with striatal cells and GDNF share the same signaling pathway to promote the extension of dopaminergic neurites. We did not detect GDNF in the CM derived from paired-cultures of mesencephalic and striatal cells (data not shown).

GDNF exert its biological effects via GDNF family receptor-α1 (GFRα1) and RET tyrosine kinase^24^. In addition to RET, GFRα1 directly interacts with NCAM and integrin β1^12^. Notably, NCAM and integrin αV participate in GDNF-induced dopaminergic neurite outgrowth^11^,^13^. Our pharmacological analysis revealed that integrin, rather than NCAM, participated in dopaminergic neurite outgrowth to the striatal cell region. Integrins are αβ heterodimers, and to date, 8β subunits have been reported to assemble with 18α subunits to form 24 distinct integrins^25^. Among them, the RGD-binding integrins (α5β1, α8β1, αVβ1, αVβ3, αVβ5, αVβ6, αVβ8, and αIIbβ3) share the ability to recognize ligands containing an RGD tripeptide active site. We also clarified the involvement of integrin α5β1 in dopaminergic neurite outgrowth to the striatal cell region using specific pharmacological inhibitors. The dopaminergic neurites preferred to extend on a fibronectin matrix. These results suggest that integrin α5β1 expression on dopaminergic neurons plays an important role in dopaminergic neurite outgrowth to the striatal cell region. However, the involvement of other integrins cannot be excluded because dopaminergic neurites also showed extension on a collagen matrix. In accordance with a previous report in which integrin α5 and β1 mRNAs were expressed in the substantia nigra and the ventral tegmental area^26^, we confirmed integrin α5β1 mRNA expression in mesencephalic cultures and detected the expression of the corresponding proteins on almost all dopaminergic neurons. Our previous study^10^ reports that the percentage of dopaminergic neurons that express G-protein-activated inwardly rectifying potassium channel (GIRK)-2 and calbindin were 48% and 46%, respectively, in this cultures. Although mesencephalic dopaminergic neurons could be divided into three distinct nuclei such as A8, A9 and A10^27^, expression of integrin α5β1 on dopaminergic neurons seems to be not restricted to GIRK-2-positive A9 cells in the substantia nigra. These findings suggest that integrin α5β1 expression on dopaminergic neurons does not necessarily participate in axonal guidance of the nigrostriatal dopaminergic projection.

Importantly, integrin α5 and β1 mRNAs were also expressed in striatal cultures. To clarify the involvement of integrin α5β1 expression on dopaminergic neurons, we selectively knock downed integrin α5 in mesencephalic and striatal cultures using a genetic-based approach. Integrin α5 assembles only with integrin β1, whereas integrin β1 assembles with integrin α1, 2, 3, 4, 5, 6, 7, 8, 9, 10, 11, V^25^. That is why knockdown of integrin α5 is necessary and sufficient to suppress the function of integrin α5β1. Knockdown of integrin α5 in dopaminergic neurons suppressed neurite outgrowth to the striatal cell region. The knockdown of integrin α5 in non-dopaminergic mesencephalic and striatal cells had no effect on neurite outgrowth. Therefore, we conclude that integrin α5β1 expression on dopaminergic neurons plays an important role in neurite outgrowth to the striatal cell region.

To enhance the dopaminergic neurite outgrowth on striatal neurons, we attempted to overexpress integrin α5 in dopaminergic neurons. Because of the size of integrin α5 coding sequence, the lentiviral titer of integrin α5-expressing vector was much lower than that of control vectors. The difference in viral titers made it difficult to equalize the infection efficiency in dopaminergic neurons of mesencephalic cultures. Therefore, we transduced mouse ES cells to stably express integrin α5 using lentiviral vectors. The elongation factor-1α (EF-1α) promoter was reported to be ineffective in TH-positive cells differentiated from ES cells^28^. However, we showed that expression of transgenes under the control of the EF-1α promoter, although reduced, was maintained in dopaminergic neurons derived from ES cells in some clones. However, when integrin α5-overexpressing ES cells were differentiated with the SDIA method, a large number of TuJ1-negative colonies appeared, thus resulting in a reduction in neural differentiation efficiency. This effect may be due to the promotion of endodermal differentiation of ES cells by integrin α5β1^29^,^30^. Regardless, we successfully prepared integrin α5-overexpressing dopaminergic neurons differentiated from ES cells. Furthermore, we showed that overexpression of integrin α5 enhanced dopaminergic neurite outgrowth on striatal cells. In support of our finding that integrin α5 plays a role in neurite outgrowth, expression of integrin α5 in neuron-like cells conferred neurite outgrowth on fibronectin^31^. Myosin light chain kinase can function downstream of integrin activation via extracellular signal-regulated kinase^32^. It is proposed that retrograde actin flow driven by myosin, which is activated by myosin light chain kinase, participates in axonal elongation^33^.

The ligands for integrin α5β1 expressed on striatal neurons remain unknown. Dopaminergic axons originating from grafts run parallel to the neurites of striatal medium-sized spiny neurons, which are the target cells for dopaminergic innervation^34^. This finding suggests the existence of specific adhesion molecules on striatal neurons for dopaminergic innervation. Recently, it was reported that integrin α5 is highly expressed in striatal neurons innervated by nigral dopaminergic neurons^35^. However, integrins are known to form heterophilic interactions at cell-cell adhesions. Fibronectin, which is the most recognized ligand for integrin α5β1, is mainly produced in astrocytes and fibroblasts^36^. Fibronectin expression is increased by cytokines and injury^37^. Moreover, the L1 cell adhesion molecule, a disintegrin and metalloproteinases-15 and -17 functionally interact with integrin α5β1^18^,^38^,^39^. Especially, L1 is reported to enhance dopaminergic neurite outgrowth in mesencephalic cultures^40^. Further investigations are needed to elucidate the integrin α5β1 ligands expressed on striatal neurons.

In summary, we showed that integrin α5β1 expression on dopaminergic neurons plays a role in the dopaminergic neurite outgrowth on striatal neurons. The present study aids in the understanding of how axons extend to their target areas during development. In addition, our findings suggest that integrin α5 overexpression in dopaminergic neurons promote the neurite outgrowth on striatal neurons.

## Materials and Methods

### Materials

Human GDNF was purchased from Alomone Labs (Jerusalem, Israel). The neural cell adhesion molecule (NCAM) function-blocking antibody (AB5032) was purchased from Merck Millipore (Billerica, MA, USA). RGDS was purchased from Peptide Institute (Osaka, Japan). RGES was purchased from Abbiotec (San Diego, CA, USA). Cyclo(RGDfV) was obtained from ENZO Life Sciences (Farmingdale, NY, USA). ATN-161 (Ac-PHSCN-NH_2_) and A5-1 (VILVLF) were synthesized by Medical & Biological Laboratories (Nagoya, Japan). The anti-integrin α5 function-blocking antibody (HMa5-1) was purchased from Santa Cruz (Dallas, TX, USA). The anti-integrin β1 function-blocking antibody (Ha2/5), fibronectin (human), collagen I (rat), and laminin (mouse) were purchased from Becton-Dickinson (Franklin Lakes, NJ, USA).

### Paired-cultivation of mesencephalic cells with striatal cells

Primary ventral mesencephalic and striatal cells were prepared from rat embryos on the 16th day of gestation. As previously described^10^,^41^, mesencephalic cells were adjacently paired-cultured with striatal cells using a water-repellent isolation wall. Briefly, the isolation wall (1.0-mm thick) was placed on a polyethylenimine-coated coverslip in a 35-mm culture dish. The mesencephalic cell suspension (120 μL) was plated inside the isolation wall (3.0 × 10^5^ cells/cm^2^), and the striatal cell suspension (1.5 mL) was plated outside the isolation wall (3.0 × 10^5^ cells/cm^2^). The isolation wall was removed 24 h after plating. Cultures were maintained in Eagle’s minimum essential medium containing 10% fetal calf serum (1–4 days *in vitro*) or horse serum (5–12 days *in vitro*). Treatment with drugs was performed after removal of the isolation wall. Primary striatal astrocytes were prepared and enriched from rat embryos on the 16th day of gestation according to previously described procedures^42^. Cultures were incubated at 37 °C in an atmosphere of 5% CO_2_ in air with 100% relative humidity. All animals were treated in accordance with the guidelines of the Kyoto University Animal Experimentation Committee and the Japanese Pharmacological Society. This study was approved by Kyoto University Animal Experimentation Committee.

### Induction of neural differentiation of mouse embryonic stem (ES) cells

Undifferentiated mouse ES cells (EB5, kindly provided by Dr. Hitoshi Niwa, RIKEN Center for Developmental Biology; Kobe, Japan) were maintained on gelatin-coated dishes in Glasgow minimum essential medium supplemented with 1% fetal calf serum, 5% knockout serum replacement (Thermo Fisher Scientific, Waltham, MA, USA), 2 mM glutamine, 0.1 mM nonessential amino acids, 1 mM pyruvate, 0.1 mM 2-mercaptoethanol, and 2,000 U/ml leukemia inhibitory factor (Nacalai Tesque, Kyoto, Japan). EB5 cells carry the blasticidin S-resistance gene driven by the Oct3/4 promoter (active in the undifferentiated state) and were maintained in medium containing 20 μg/ml blasticidin S to eliminate differentiated cells^43^,^44^. Dopaminergic neurons were differentiated from mouse ES cells using the SDIA method as previously described^19^. Briefly, ES cells were co-cultured on PA6 stromal cells at a single-cell density in differentiation medium (Glasgow minimum essential medium supplemented with 10% knockout serum replacement, 2 mM glutamine, 0.1 mM nonessential amino acids, 1 mM pyruvate, and 0.1 mM 2-mercaptoethanol). For the replating of ES cell-derived cells on striatal cells, ES cell-derived colonies were detached from PA6 cells after 13 days of differentiation by incubation with collagenase type IV (Sigma-Aldrich, St. Louis, MO, USA) for 3 min at 37 °C. The detached colonies were isolated by spontaneous sedimentation, and gently triturated into single cells in the presence of Dispase (Thermo Fisher Scientific). ES cell-derived cells were replated on striatal cultures in the differentiation medium.

### Immunocytochemistry

Immunocytochemistry was performed as previously described^10^. Following fixation with 4% paraformaldehyde, cells were sequentially incubated with 0.2‒0.5% Triton X-100, primary antibodies, and secondary antibodies. For diaminobenzidine staining, cells were incubated with the avidin–biotinylated horseradish peroxidase complex (Vector Laboratories, Burlingame, CA, USA) and reacted with a diaminobenzidine solution (Dojindo Laboratories, Kumamoto, Japan). For nuclear staining, cells were incubated with Hoechst 33258 (100 μg/mL). The following antibodies were commercially obtained: anti-TH from Merck Millipore, anti-MAP2 from Sigma-Aldrich, anti-pNF (SMI 31) and anti-neuronal class III β-tubulin (Tuj1) from Covance (Princeton, NJ, USA), anti-GFP from Nacalai Tesque, anti-GFAP from Merck Millipore, anti-integrin α5 (HMa5-1) and anti-integrin β1 (N-20) from Santa Cruz. Images of living or immunostained cells were obtained using an IX81 inverted research microscope (Olympus, Tokyo, Japan), a BZ-8100 fluorescence microscope (Keyence, Osaka, Japan), and an A1RMP multiphoton confocal microscope (Nikon, Tokyo, Japan). The fluorescence of immunostained cells was analyzed on a BD FACSAria III cell sorter (Becton-Dickinson).

### Measurement of dopaminergic neurite length

To measure dopaminergic neurite length in the paired-cultures of mesencephalic and striatal cells, cultures were processed for TH immunostaining. As previously described^10^, the borderline of the mesencephalic cell region was defined as the position of the most laterally located TH-positive dopaminergic soma. The distance from TH-positive dopaminergic growth cones to the borderline was measured and defined as the neurite length. The sum of all dopaminergic neurite lengths in a width of 890 μm was calculated. To produce a continuous image, several photographs were merged using a BZ Analyzer (Keyence). There were four subjects for each experiment.

The measurements of neurite length of ES-derived dopaminergic neurons were completed using Image J software (National Institutes of Health, Bethesda, MD, USA) with the NeuronJ plug-in.

### RT-PCR

Total RNA was isolated using a High Pure RNA Isolation Kit (Roche, Basel, Switzerland) according to the manufacturer’s instructions. Total RNA was reverse-transcribed using oligo (dT) primers and RT-PCR was performed using a PrimeScript RT-PCR Kit (Takara Bio, Shiga, Japan) according to the manufacturer’s instructions. Table 1 shows the primer sets used for amplication. The PCR conditions included 30 cycles of 30 s at 94 °C, 30 s at 60 °C, and 30 s at 72 °C.

### Generation of lentiviral vectors and *in vitro* transduction

Plasmids, which were required to generate the third-generation self-inactivated human immunodeficiency virus-1–based lentiviral vectors, were kindly provided by Dr. Hiroyuki Miyoshi (RIKEN BioResource Center). Integrin α5 shRNA was prepared by annealing 67 base pair sense and antisense oligos that contained a 19-base stem from the rat integrin α5 sequence (5′-CACTAGCCAACCAGGAGTA-3′)^45^ and a 15-base loop (5′-ACGTGTGCTGTCCGT-3′). Control shRNA contained a non-targeting stem (5′-ACGTGACACGTTCGGAGAA-3′). The annealed oligos were subcloned into pENTER-H1 at the BglII and XbaI sites, and were recombined into CS-RfA-EVBsd using Gateway LR Clonase II (Thermo Fisher Scientific). Mouse integrin α5 cDNA was amplified by PCR from mouse whole brain cDNA, and the coding region was verified by DNA sequencing. The cDNA was subcloned into CSII-EF-RfA-IRES2-Venus by replacing the RfA Gateway cassette. The vector contains an EF-1α promoter and an internal ribosomal entry site 2 (IRES2) followed by Venus, which is a variant of yellow fluorescent protein^46^. The lentiviral vectors expressing Venus only or integrin α5 followed by Venus were generated by transient cotransfection of HEK293T cells with CSII-EF-IRES2-Venus or CSII-EF-Itga5-IRES2-Venus, respectively, the packaging construct (pCAG-HIVgp), and the envelop- and Rev-expressing construct (pCMV-VSV-G-RSV-Rev). Two days after transfection, the vector-containing supernatant was collected, filtered through a 0.22-μm-pore-size filter, and concentrated by centrifugation at 50,000 g for 2 hours at 20 °C. The virus pellet was resuspended in culture medium and stored at −80 °C until use. For the enhancement of lentiviral infection, polybrene (8 μg/mL) was added to the lentiviral vector-containing medium.

### Western blot analysis

The Western blot analysis was conducted as previously described^47^, except that the detection of integrin α5 was performed under non-reducing conditions. The following antibodies were commercially obtained: anti-integrin α5 (AB1928) from Merck Millipore and anti-β-actin (AC-15) from Sigma-Aldrich.

### Statistics

The statistical significance of the differences between three or more groups was analyzed with a one-way analysis of variance (ANOVA) followed by post-hoc multiple comparisons using the Turkey’s test. Statistical significance was defined as *p* < 0.05. Data are expressed as the mean ± standard error of the mean (SEM).

## Additional Information

**How to cite this article**: Izumi, Y. *et al*. Integrin α5β1 expression on dopaminergic neurons is involved in dopaminergic neurite outgrowth on striatal neurons. *Sci. Rep.* **7**, 42111; doi: 10.1038/srep42111 (2017).

**Publisher's note:** Springer Nature remains neutral with regard to jurisdictional claims in published maps and institutional affiliations.

## Figures and Tables

**Table 1:** Primer sets used in PCR analysis for mRNA expression.

Gene name	Amplicon size (bp)	Primer sequences
Integrin α1	115	5′-ATGCCTTGCGTGAAGTTGG-3′
		5′-GAAATCCTCCATTCGGGTTG-3′
Integrin α2	101	5′-TTGCTGTTGGCTATGGTTGC-3′
		5′-TCTGTGGTCTCGTCCGTCTC-3′
Integrin α4	112	5′-CGAGTTTCAAGCAGTGGAGAG-3′
		5′-GATGCCCAAGGTGGTATGTG-3′
Integrin α5	99	5′-GCTGCATTTCCGAGTCTGG-3′
		5′-TAGGGCATCTTCAGGGCTTC-3′
Integrin α6	120	5′-GAGCTCCCTATGACGATCTGG-3′
		5′-CAGCGATTGAGTAGCCGAAG-3′
Integrin α7	127	5′-CAGGCAGATGGGGATGATG-3′
		5′-AGGCATTCTCGTTGGACAGG-3′
Integrin α8	104	5′-AGGCCAGAGCATTTCAAATACAG-3′
		5′-GGTGGTTGTGGAGGAAGAGG-3′
Integrin αV	119	5′-TTAAGACGCCCGAAAAGAATG-3′
		5′-AGCAGTTCCACAGCCCAAAG-3′
Integrin β1	139	5′-GACCTGCCTTGGTGTCTGTG-3′
		5′-GGGCAACTTCTCCCTGCTTT-3′
Integrin β2	118	5′-TGGTGAGAAGCAGGCAGAGA-3′
		5′-TACCTCAGGGAGGGATGTGG-3′
Integrin β3	123	5′-GGACGGAAACGCTTACAACATC-3′
		5′-ACCAGGAGGAGGACACCAATC-3′
Integrin β4	145	5′-AAACTGCAAGGAGAACGCATC-3′
		5′-AGGCAGACTCGGTGGAGAAC-3′
Integrin β5	95	5′-TTCCCCAACTGTGTCCCTTC-3′
		5′-ACCCTCTGCTTCCTCACTTCC-3′
Nurr1	253	5′-TGAAGAGAGCGGACAAGGAGATC-3′
		5′-TCTGGAGTTAAGAAATCGGAGCTG-3′
Ptx3	373	5′-AGGACGGCTCTCTGAAGAA-3′
		5′-TTGACCGAGTTGAAGGCGAA-3′
GAPDH	452	5′-ACCACAGTCCATGCCATCAC-3′
		5′-TCCACCACCCTGTTGCTGTA-3′
